# Subfamily specific conservation profiles for proteins based on n-gram patterns

**DOI:** 10.1186/1471-2105-9-72

**Published:** 2008-01-30

**Authors:** John K Vries, Xiong Liu

**Affiliations:** 1 Department of Computational Biology, School of Medicine, University of Pittsburgh, Pittsburgh, PA 15213, USA

## Abstract

**Background:**

A new algorithm has been developed for generating conservation profiles that reflect the evolutionary history of the subfamily associated with a query sequence. It is based on n-gram patterns (NP{*n,m*}) which are sets of *n *residues and *m *wildcards in windows of size *n+m*. The generation of conservation profiles is treated as a signal-to-noise problem where the signal is the count of n-gram patterns in target sequences that are similar to the query sequence and the noise is the count over all target sequences. The signal is differentiated from the noise by applying singular value decomposition to sets of target sequences rank ordered by similarity with respect to the query.

**Results:**

The new algorithm was used to construct 4,248 profiles from 120 randomly selected Pfam-A families. These were compared to profiles generated from multiple alignments using the consensus approach. The two profiles were similar whenever the subfamily associated with the query sequence was well represented in the multiple alignment. It was possible to construct subfamily specific conservation profiles using the new algorithm for subfamilies with as few as five members. The speed of the new algorithm was comparable to the multiple alignment approach.

**Conclusion:**

Subfamily specific conservation profiles can be generated by the new algorithm without aprioi knowledge of family relationships or domain architecture. This is useful when the subfamily contains multiple domains with different levels of representation in protein databases. It may also be applicable when the subfamily sample size is too small for the multiple alignment approach.

## Background

Protein homologs are amino acid sequences with a common evolutionary ancestor. Substitutions, insertions and deletions over the course of evolutionary time cause the patterns of residues and gaps in homologs to drift away from each other [1,2]. Conservation profiles are a measure of the shared patterns that remain. The conserved regions revealed in profiles are useful for identifying sites that are important for structure and function [3]. Traditionally they have been constructed from multiple sequence alignments (MSA) using scoring matrices and weighted averages [4]. This approach yields a consensus profile that is a function of the sequence sample in the multiple alignment. It also requires a chain of assumptions that can be problematic. There are many ways to generate scoring matrices and these matrices vary in their sensitivity to remote homologs [5-8]. Many proteins contain multiple domains or overlapping and/or nested domains that strongly influence alignment [9]. Sequences for multiple alignments often require preprocessing to eliminate low complexity regions [10]. The protein sequence samples available for multiple alignment are frequently skewed requiring the application of weighting algorithms [11]. Finally, multiple alignment requires a parameterized gap penalty [12].

A new algorithm for generating sequence specific conservation profiles has been developed that avoids the assumptions associated with the MSA approach. It is based on n-gram patterns (NP{*n,m*}) which are sets of n residues and m wildcards in windows of size *n+m *that start with a residue.

Interest in these patterns was sparked by the success of an alignment-independent protein classification algorithm based on the distribution of NP{4,2} patterns [13]. A study was conducted comparing the classification results obtained using 4-grams in windows of 5 or 6 with published results obtained using PSI-BLAST [12]. Unpublished classification runs using windows of 7 with 3 gaps were also performed. Classification runs with 4-grams alone or with windows of 5 with 1 gap were not as effective as windows of 6 with 2 gaps. Increasing the size of the window to 7 with 3 gaps showed no further improvement in classification accuracy. It was also possible to lower the combinatoric complexity associated with NP{4,2} patterns by eliminating the wildcard in the first position without adversely affecting the results. Features of interest in NP{4,2} patterns included: (1) the inclusion of all possible n-gram combinations for 1 ≤ n ≤ 4; (2) a window wide enough to capture alpha helix and beta sheet related periodicities for 2 ≤ n ≤ 5; (3) an implied scoring matrix due to the presence of wildcards at variable positions; (4) a low probability for finding redundant n-gram patterns in the same sequence; (5) a high probability of family membership for two sequences that contain the same pair of non-overlapping NP{4,2} patterns; and (6) the existence of all theoretically possible NP{4,2} patterns in nature [13,14]. The new algorithm generates conservation profiles by exploiting the difference in the distribution of NP{4,2} patterns between family and non-family members. Family membership is determined without apriori knowledge by analyzing the covariance of NP{4,2} pattern counts in sequence samples with progressively increasing degrees of similarity. Samples with low degrees of similarity contain mostly noise. Samples with high degrees of similarity contain mostly family members. This results in two variance patterns that can be separated using singular value decomposition (SVD). Separate reconstruction of the traces using the first and second eigenvectors provides an effective filter for detecting the weak signal generated by a small number of family members in a randomly distributed set of non-family members.

## Results

### Theoretical background

Let us define an n-gram pattern (NP{*n,m*}) in a protein sequence to be a set of *n *specific residues and *m *wildcards (gaps) in a window of size *w *where *w = n+m*. Let us represent the amino acid patterns in protein sequences as collections of overlapping n-gram patterns. If we add the constraint that an n-gram pattern must not begin with a wildcard in order to reduce the problem of combinatorics, each position in a protein sequence contains *(w-1)!/((w-1)-(n-1))!(n-1)! *unique n-gram patterns. If the sequence is also a member of a protein family that descended from a common evolutionary ancestor it will share a portion of its n-gram patterns with family members. This will lead to an n-gram pattern distribution for the family that differs from the distribution over the protein universe. Let us define the conservation profile for such a sequence as the ratio of the average n-gram pattern count at each position over its family members to the average n-gram pattern count over the protein universe. When there is apriori knowledge of family membership, determining the n-gram pattern conservation profile for a sequence is reduced to a straight-forward counting task. When family membership is not known a different approach is required.

If the n-gram patterns at each position in a query sequence are counted over the protein universe, a profile is obtained that is a mixture of signal and noise. The signal reflects the expectation for an n-gram pattern at each position given that it is a member of a protein family. The noise reflects the expectation for the same n-gram pattern over all sequences. It would be possible to obtain the conservation profile for the query sequence from the counts over the protein universe if the signal could be separated from the noise. This can be accomplished by separating the sequences in the protein universe into samples (bins) based on similarity to the query sequence. Bins with low degrees of similarity will contain mostly noise. Bins with high degrees of similarity will contain mostly signal. Since noise is randomly distributed, the covariance between signal and noise will be low. If a covariance matrix were generated from the bins, singular value decomposition [15] of the matrix should separate the variance into a set of eigenpairs reflecting mostly signal and a set of eigenpairs reflecting mostly noise. In such a case, conservation profiles could be obtained by reconstructing the samples with the eigenpairs reflecting predominantly signal.

The strength of a signal is a function of the size of its family and the degree of conservation of each n-gram pattern. For bins reconstructed from eigenpairs reflecting signal variance, the average amplitude should increase as the percentage of family members increases. The average amplitude of reconstructions from eigenpairs reflecting noise variance should go in the opposite direction. If the signal from the protein family were significantly stronger than the noise, it should appear in the first eigenpair.

**Figure 1:**
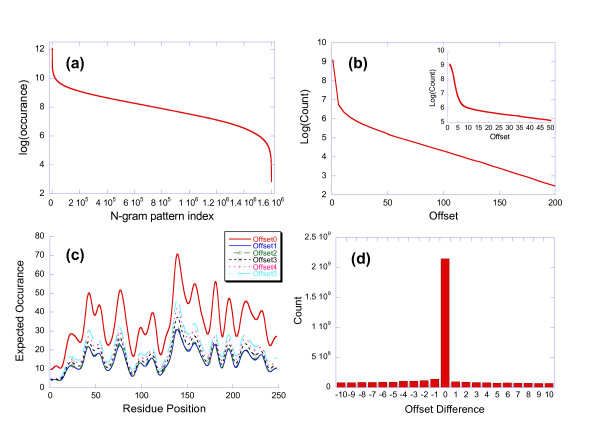
(a) Logarithmic histogram of NP{4,2} patterns in UniProt. All theoretical NP{4,2} patterns are present. The distribution is relatively flat over the majority of the range. (b) Histogram of the overlap between shared NP{4,2} patterns. The majority of patterns have an overlap of 5 which decreases exponentially as the degree of overlap approaches 0. (c) Noise level for carbonic anhydrase (P00918). The y-axis represents the expectation by random chance in the SPT data set for pairs of NP{4,2} patterns with overlaps ranging from 0–5. The noise level decreases significantly for overlapping pairs compared to NP{4,2} patterns by themselves (overlap = 0). (d) Distribution of offset differences between shared NP{4,2} patterns in different sequences. More than 80% of shared NP{4,2} pairs have an offset of 0. The remainder are distributed in a random fashion over the range of possible offsets. Pairs of shared NP{4,2} patterns with zero offset represent n-gram pattern local alignments (NPLAs).

We can parse any query sequence into its constituent n-gram patterns by advancing a window of size *w *along the sequence one residue at a time. From a study of the statistical properties of NP{*n,m*} patterns for 1 ≤ *n *≤ 5 and 0 ≤ *m *≤ 3 in the UniProt database [16] or the SPT data set (~2.1 M sequences), it is apparent that the useful range for *n *and *m *is very narrow. For all observed values of *n*, the noise level is roughly proportional to the product of the probability of its residue elements. This implies that the noise level decreases as the value of *n *increases. Unfortunately, raising *n *to values higher than 4 results in NP{*n,m*} sample sizes that are too small for statistical analysis. The value for *m *has little effect on the noise level. Increasing the value of *m *improves the ability to recognize patterns with gaps which is offset by an increase in combinatorial complexity. The presence of a variable gap creates an implied scoring matrix. A good compromise for the *n *and *m *parameters appears to be NP{4,2}. The distribution of NP{4,2} patterns is shown in Figure 1a. The plot shows that all possible NP{4,2} patterns (20^4*10), 1.6 M) occur in UniProt [16]. The sample sizes range from ~180,000 for the most common patterns to ~20 for the rarest patterns. The majority of patterns have a flat distribution with a sample size of ~4,000.

Since the strength of NP{4,2} conservation signals in a query sequence is a function of the size of the family and the degree of conservation at each position, families with more than 100 members and degrees of conservation greater than 25% have expected counts that are significantly greater than the counts expected for noise. Unfortunately, more than 2/3 of protein families have less than 100 members and degrees of conservation may be less than 25% [17]. One way to enhance the signal to noise ratio for these smaller families is to analyze shared pairs of NP{4,2} patterns. This increases the effective value of the *n *parameter to 5–8 depending on the degree of overlap. Allowing a variable gap between a pair of NP{4,2} patterns also extends the effective range of the *m *parameter. The distribution of the overlap between shared pairs of NP{4,2} patterns in the SPT data set is shown in Figure 1b. The highest probability is associated with an overlap of 5. This drops exponentially to a very small probability as the overlap approaches 0. This is demonstrated in Figure 1c which treats shared NP{4,2} pairs as events in a Poisson distribution [18]. The y-axis in this figure shows the expectation by random chance for the overlapping pairs in carbonic anhydrase (P00918) over the 2,128,677 sequences in the SPT data set for overlaps ranging from 0–5. The highest noise level (expectation by random chance) occurs when the overlap is zero which is equivalent to the NP{4,2} pattern by itself. The figure shows that the noise level drops as the effective value of the *n *parameter increases.

Let us define the offset between shared pairs of NP{4,2} patterns to be the absolute difference between the starting positions of the NP{4,2} pairs in each sequence. If the offset between shared sequences were a random event, it should reflect the distribution of the NP{4,2} pattern overlaps depicted in Figure 1b. Figure 1d which plots the offsets for NP{4,2} shared pairs from 100 randomly selected sequences from the SPT data set shows that more than 80% of the pairs have the same offset. This implies that the majority of shared NP{4,2} patterns form a fixed local alignment (NPLA). This suggests that sequence comparison based on pairs of NP{4,2} patterns with fixed offsets (NPLAs) should work better than pairs of patterns with variable offsets since NPLAs have a higher information content (higher signal to noise ratio in this context). The algorithm described in the next section generates conservation profiles for query sequences by counting the common elements in the NPLAs shared by family members. It identifies family membership by applying singular value decomposition to the covariance matrix created from samples of the protein universe with progressively increasing degrees of similarity to the query.

### The NPLA algorithm

**Figure 2:**
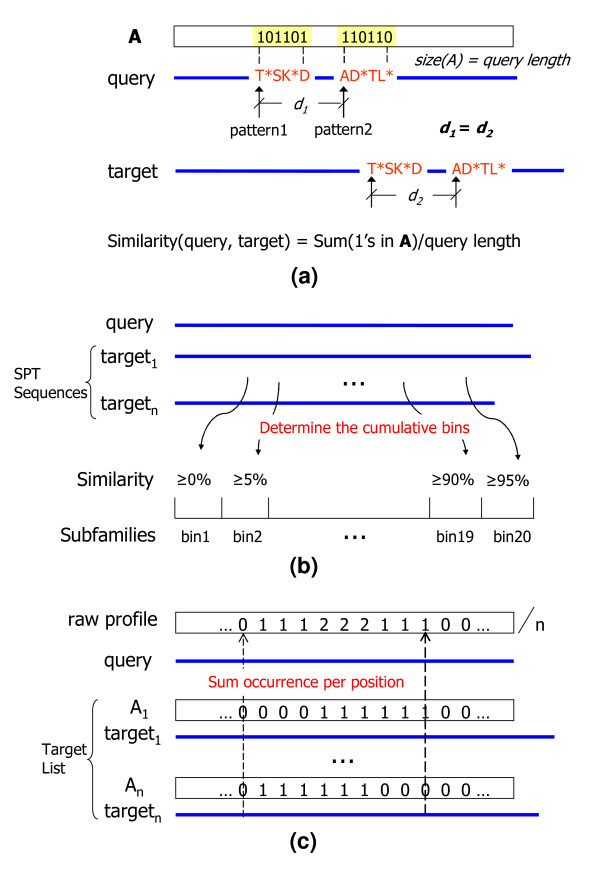
Initial steps in the NPLA algorithm: (a) For each shared pair of zero-offset NP{4,2} patterns between a query sequence and a target sequence, the non-wildcard positions in a collection sequence equal in length to the query sequence are set to 1. (b) The target sequences are divided into 20 sets (bins) based on the similarity of their NP{4,2} pattern content. (c) Raw conservation profiles are generated for each similarity bin by summing over the collection sequences associated with the bin and dividing by the number of members in the bin.

The goal of the NPLA algorithm is to generate a conservation profile that is specific to a given query sequence when the family membership of the sequence is unknown. The algorithm is written in Java v1.5.0 [19]. The first step is to identify and count the non-wildcard positions in the NPLAs shared by the query sequence and the ~2.1 M target sequences in the SPT data set. This process is illustrated in Figure 2a. A collection sequence equal in length to the query sequence is initialized to zero for each target sequence. The non-wildcard position in the collection sequence for each common non-wildcard element in shared NPLAs is set to 1. The combinatorics associated with NP{4,2} patterns generates 10 different patterns for each position in the query sequence. These patterns are tested in an order that favors the longest contiguous residue runs. The algorithm stops when the first pair of NP{4,2} patterns is found. This avoids double counting for the pattern and it finds the longest contiguous runs of residues in common. This provides an implicit substitution matrix and it insures that each position is counted only once. Summing the 1s for each collection sequence also provides a measure of similarity with respect to the query sequence for each target sequence. The similarity threshold with respect to the target sequence is used as the basis for separating the SPT data set into 20 samples with increasing levels of identity. This process is illustrated in Figure 2b. The 95% bin for example represents all target sequences with 95% or greater similarity. The 0% bin represents all sequences in the SPT data set. The collection sequences in each subset are then summed and normalized with respect to sample size to provide 20 raw conservation profiles. This is process is shown in Figure 2c.

**Figure 3:**
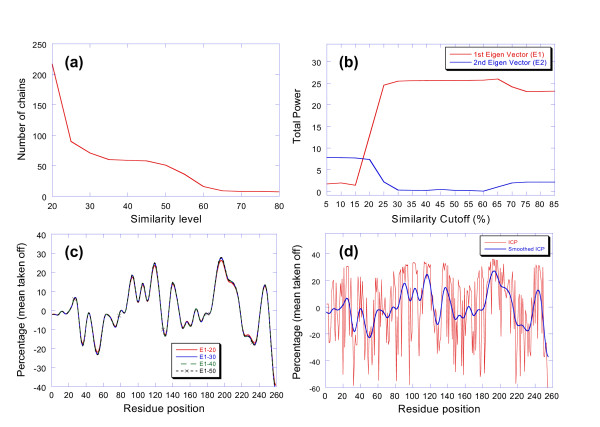
(a) Distribution of the similarity threshold samples for carbonic anhydrase (P00918) over the range from 20–80%. The sample contains over 200 members at the 20% level. (b) Plots of the average amplitude of reconstructions from the first and second eigenvectors for P00918. The average amplitude for the reconstruction from the first eigenvector goes from low to high as the similarity to the query increases. The reconstruction from the second eigenvector goes in the opposite direction. The amplitude is relatively flat over the 20–80% range. (c) Plot of the reconstructions using the first eigenvector for similarity ranges from 20–60%. The profile is almost invariant over this range. (d) Invariant conservation profile (ICP) for P00918 reconstructed from the 40% similarity level. The plot is shown with and without nearest-neighbor smoothing.

A covariance matrix is generated from the 20 raw profiles, where the percentage value or Y value of each profile is treated as a random variable. The matrix is then subjected to singular value decomposition. Reconstructions of the 20 raw profiles are generated for each individual eigenvector with a significant eigenvalue (> 0.01). The applicability of the algorithm is then assessed based on the sample and eigenvalue distribution and the amplitude profiles of the reconstructions. Four criteria are employed: (1) does the sample have an adequate size over a contiguous range of bins ? (2) are there two dominant eigenvalues that account for the majority of the variance (> 0.90) ? (3) does the amplitude profile of the reconstruction from the first eignevector go from low to high as sample similarity increase ? and (4) does the reconstruction from the second eigenvector go in the opposite direction ? The carbonic anhydrase profile (P00918) from the Pfam-A family PF00194 is characteristic of the sequences that meet these criteria. The sample distribution for similarity thresholds ranging from 15% to 95% is shown in Figure 3a. There is a progressive rise in the degree of similarity over the range and the samples are large enough for statistical analysis. The percentage of variance associated with the first two eigenvalues is 0.75 and 0.20. A plot of the amplitude of the samples reconstructed from the first two eigenvectors is shown in Figure 3b. The average amplitude of the reconstruction from the first eigenvector goes from low to high as the percentage of family members in the sample increases. The reconstruction from the second eigenvector goes in the opposite direction. It can also be seen that the amplitude of the reconstruction from the first eigenvector is invariant over the central part of the similarity range. Reconstructions of the raw profiles using the first eigenvector are shown in Figure 3c for similarity thresholds varying from 20–60%. The final plots have been subjected to 16 iterations of nearest-neighbor smoothing to eliminate high frequency noise [20]. The conservation profiles over this range are nearly invariant. The final invariant conservation profile (ICP) is selected from this set by identifying the profile with the least rmsd difference with its neighbors. The ICP trace representing the 40% similarity level is shown in Figure 3d with and without smoothing.

### Computational efficiency

**Figure 4:**
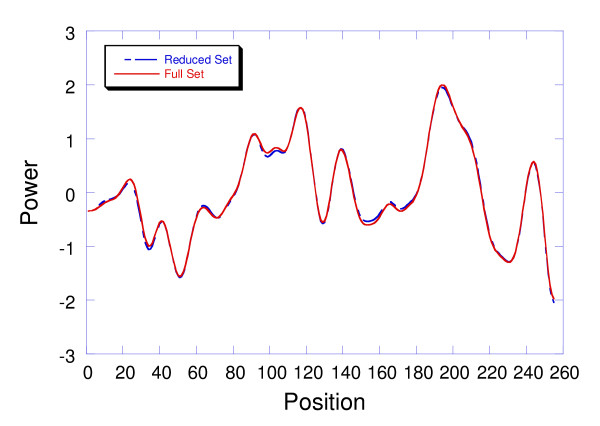
Comparison of the invariant conservation profile (ICP) for P00918 generated from the reduced SPT data set (~40 K) with the ICP generated from the full SPT data set (~2.1 M). The reduced SPT set was created from an inverted index of trigrams. The rmsd difference between the traces is only 0.042. This was typical for the 4,248 traces in the SPT data set.

Comparison of the NP{4,2} patterns in a query sequence with all sequences in the SPT data set (~2.1 M) is costly from the computational standpoint. Profile generation using a single processor requires 45–60 minutes. If the average Pfam-A family only has 50–100 members, most of the processed sequences represent noise. Since noise is distributed randomly, its variance should not change as long as the sample is large enough from a statistical point of view. If most of the sequences representing noise could be eliminated up front, the computational time would decrease significantly without affecting the variance associated with the signal eigenvector (first eigenvector). Using the multiple alignments for the 8183 families in Pfam-A, we determined the probability of finding at least one n-gram (contiguous run of *n *residues) in common between all family members for 1 ≤ *n *≤ 4. It should be noted that this process was not related to NP{4,2} patterns. The objective was to lower the computational burden by eliminating up front a significant percentage of the sequences that had no chance of containing family members. The idea was to find an n-gram that was present in all family members and to eliminate processing on all sequences that did not contain that n-gram. The size of the n-gram was critical because smaller n-grams are more likely to be found in all family members, but larger n-grams were less likely to be found by random chance. The best balance was achieved with trigrams where *n *= 3. Approximately 96% of all family members over the 8183 Pfam-A families had at least one trigram in common, while the expectation of finding a trigram match by random chance in the SPT data set was approximately 275. When *n *was smaller than 3, the expectation of finding a match in the SPT data set by random chance was too high to be useful. When *n *was larger than 3, the coverage for family members was too low. An inverted index was created mapping the trigrams in the SPT data set to integers representing UniProt accession numbers. A preprocessor for queries was constructed which looked up each trigram in the query sequence while incrementing the integer position of its UniProt accession number in a collection sequence. Sorting this buffer in descending order based on trigram hits and taking the first 40,000 members (top 2%) captured the majority of the family while eliminating 98% of the noise. This decreased the processing time by two orders of magnitude. Computational times of approximately 1 minute were achieved on conventional CPUs. Comparison of profiles generated with the complete and reduced SPT data set over a selection of 4,248 queries showed an average rmsd difference of less than 5%. This is illustrated for the P00918 profile in Figure 4. The example shown here is typical for all the ICPs generated from the SPT data set. The reduced SPT data was used in the studies comparing ICP conservation profiles to profiles generated from MSAs. The time complexity for the NPLA algorithm is comparable to the time complexity of the standard sequence search algorithms such as PSI-Blast. The time complexity of running PSI-BLAST on the SPT database is O(*nl*), where *n *is the number of sequences in the SPT database and *l *is the length of the longest sequence [21,22]. The time complexity of running the NPLA algorithm is O(*n'l*), where *n' *is the top 2% percent of the sequences in the SPT database.

### Comparison of the NPLA method and the consensus method

The NPLA algorithm generates a conservation profile that is different from the profile generated by MACP. The NPLA algorithm only sees family members that are related to the domains present in the query sequence. The MACP profile may see family members containing domains that are not part of the query sequence. This depends on the parameters used for multiple alignment. If the multiple alignment is constructed so that only the domains in the query sequence are represented in the alignment, the NPLA algorithm and the MACP algorithm should yield similar results. The NPLA algorithm yields a profile that is specific to the subfamily of the query sequence without requiring information about the domain and subdomain architecture of the family. The term ICP reflects that fact that the NPLA profile tends to be invariant with respect to sample size as long as five or more subfamily members are present.

To show the difference between the ICP profile and the MACP profile, the profiles from 120 randomly selected Pfam-A families were examined [17]. The Valdar-Thornton approach [4] was used to generate the MACPs. The mathematical basis for this method is outlined in the methods section. The test set was created from a list of Pfam-A accession numbers (8,183) that had been sorted into ascending order based on the number of members in each family's full alignment. Four samples of 30 accession numbers each were drawn from this list centered on N = 30, N = 100, N = 500 and N = 1000 where N was the number of sequences in the full alignment. The respective number of seed alignment sequences in each of the four samples were 203, 441, 1,666 and 1,938. Each family was clustered hierarchically according to the pairwise evolutionary distance between its family members using the approach outlined in the methods section. Subfamilies were simulated by generating MACPs from each cluster that contained the query sequence and a minimum of 20 members. Each MACP was compared to the corresponding ICP by advancing the traces against one another one position at a time and selecting the best rmsd fit. Prior to this step, the two traces were normalized for power so that each had an average power of 1.0 arbitrary units per position. The additional constraint that the shorter of the two sequences should have a minimum of 90% overlap was also enforced. The mathematical basis for the Valdar-Thornton approach, the details of the clustering approach and a table of the UniProt accession numbers used in this study are presented in the Methods Section.

**Figure 5:**
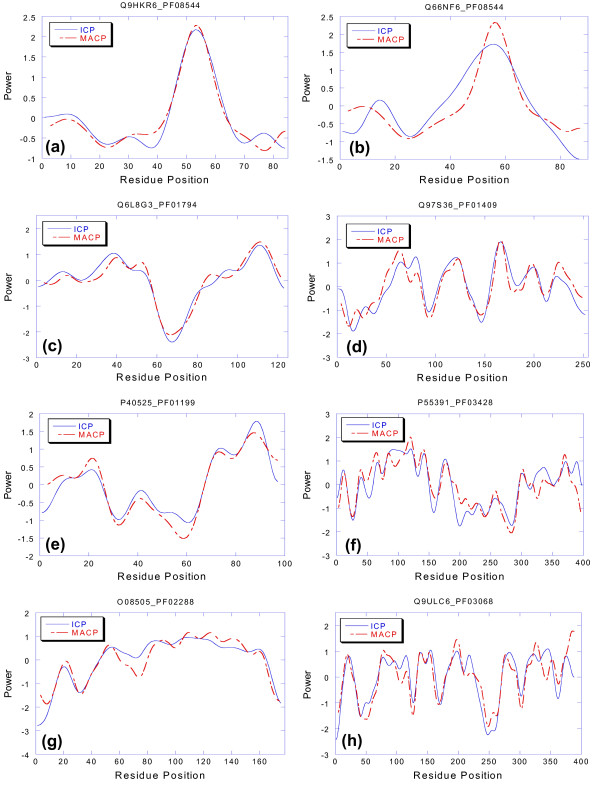
Best rmsd fits for the four family size ranges. All rmsds are two standard deviations or better below the mean. For each family range, the best fit is shown on the left and the worst fit is shown on the right. (a,b) = S1000, (c,d) = S500, (e,f) = S100 and (g,h) = S30. The major peaks in the ICP traces are very close to the peaks in the MACP traces in all cases. This shows that a significant number of examples exist (N = 103) where the ICP and the MACP are the same.

The comparison between ICP and MACP for the four samples as a function of level in the cluster hierarchy is shown in Table 1. At level 1 the multiple alignment contains all the Pfam-A family members. As the level in the hierarchy increases, the multiple alignment samples get smaller and the pairwise difference in evolutionary distance between members decreases. The N30–N1000 columns show the number of clusters at each level that contained at least 20 members. The RMSD30–RMSD1000 columns show the average rmsd difference between the ICP and MACP traces for the best rmsd fits at each level. A strong correlation between hierarchical level (i.e., pairwise consistency) and rmsd difference is apparent. The ICP and the MACP approach one another as the pairwise difference between the cluster members decreases. If the query sequence were a member of a distinct subfamily and the multiple alignment were limited to members of that subfamily, this would be the expected behavior. This table also implies that a subset of MACP traces should exist that are the same as their corresponding ICP traces. This should happen whenever the multiple alignment used to generate the MACP is dominated by a single subfamily and the query sequence used to generate the ICP is a member of that subfamily. Figure 5 shows that a significant number of examples exist in all four test samples where the ICP and MACP traces have the same peak and amplitude patterns. The rmsd difference between the ICP and the MACP was determined for each member in each of the four samples (N = 30, N = 100, N = 500, N = 1000). A list rank ordered by ascending rmsd difference was created for each sample. For each sample, representatives that were more than 2 standard deviations below the mean were selected for plotting. The left sided traces show the best fit for each sample. The right sided traces show the fit at the 2 standard deviation limit. The total number of traces more than 2 standard deviations below the mean for all 4 samples was 103.

**Table 1:** Comparison of ICP and MACP as a function of cluster level

**LEVEL**	**N30**	**RMSD30**	**N100**	**RMSD100**	**N500**	**RMSD500**	**N1000**	**RMSD1000**
**1**	203	0.999	441	1.024	1666	0.954	1938	0.977
**2**	137	0.992	434	1.007	1666	0.944	1938	0.969
**3**	29	0.908	359	0.975	1649	0.933	1933	0.958
**4**	11	0.861	232	0.929	1599	0.928	1903	0.948
**5**			145	0.911	1523	0.922	1863	0.938
**6**			103	0.910	1373	0.915	1703	0.926
**7**			80	0.941	1100	0.919	1486	0.916
**8**			39	0.831	864	0.913	1252	0.901
**9**			19	0.820	667	0.898	1032	0.890
**10**					470	0.875	862	0.893
**11**					346	0.871	672	0.878
**12**					223	0.852	465	0.870

**Figure 6:**
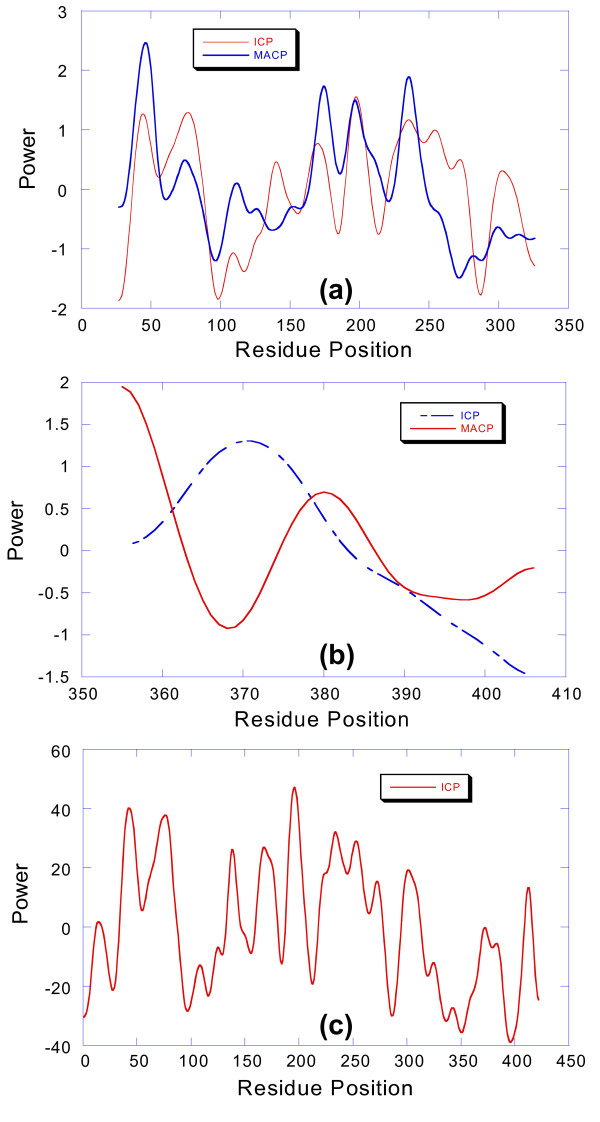
(a) ICP and MACP traces for the tRNA-synt_1b domain. The traces are correlated, but there are some differences because the ICP is specific for P22326 and the MACP reflects the consensus over the PF00579 family. (b) ICP and MACP traces for the S4 domain. These traces are not the same because P22326 has minimal representation in the MACP for PF01479 which is a consensus over 4,933 members. (c) The ICP trace for the complete P22326 sequence. Both domains as well as the interdomain regions are represented with emphasis on the subfamily associated with P22326. Note that the focus on the P22326 subfamily provides additional definition for the S4 portion of the trace.

As one moves down the rank ordered list, the rmsd difference between the ICP and the MACP steadily increases. The resemblance between the traces is still apparent as the mean is approached, but thereafter the two traces differ markedly. This difference is further compounded when the full sequence is employed for ICP generation rather than the seed alignment portion. In these cases, there may be multiple domains, overlapping domains and domains with differing counts in the SPT data set. Since the ICP does not require multiple alignment, or knowledge of domain architecture or family membership, it can generate conservation profiles in a straight-forward manner when the multiple alignment approach becomes problematic. The statistical validity of theses profiles can be determined using the approach shown in Figure 3. This is illustrated in Figure 6 using the conservation profile for P22326 (tyrosyl-tRNA-synthetase). This sequence contains a tRNA-synt_1b domain (PF00579) from a family with 1085 members [17], and an S4 domain from a family with 4,933 members. Figure 6a shows the comparison between the ICP and the MACP for the seed alignment sequence of the tRNA-synt_1b domain. The two traces have similarities but they also display significant differences. The ICP is specific to the query sequence while the MACP represents the consensus for all subfamilies in the domain. The differences are marked for the S4 domain which is shown in Figure 6b. In this case the query sequence has a limited representation in the consensus. The ICP profile for the entire sequence is shown in Figure 6c. This trace relates the two domains to one another as well as the portions of the trace not covered by the multiple alignments. The statistical basis for this trace using the criteria shown in Figure 3 remains sound.

## Discussion and Conclusion

The NPLA algorithm generates conservation profiles by treating conservation as a signal-to-noise problem where the signal is the probability of shared pairs of NP{4,2} n-gram patterns between family members and the noise is the probability of those pairs by random chance. The NPLA conservation profiles are specific to the query sequence and cover all of its residues. The conservation profiles do not require multiple alignment or explicit scoring matrices and they are not influenced by multiple, overlapping or nested domains. The NPLA conservation profiles are generated from target sequence samples containing mixtures of signal (family members) and noise (non-family members). They are invariant as long as the overall count of family members is sufficient and there is a steady progression in the similarity of family members over the target samples from low to high.

Comparison of the ICP profiles generated by the NPLA algorithm with the MACP profiles generated from multiple sequence alignment over a large sample of Pfam-A families shows that the ICP trace is nearly identical to the MACP trace whenever the query sequence is well represented in the multiple alignment used for the MACP. The degree of representation can be assessed by examining the distribution of evolutionary distances over family members and comparing that with the distribution with respect to the query sequence. Query distributions with means below the means of their families tend to have good representations. The opposite is true for query distributions with means above their family means. The absolute value of the family mean is also important. If the mean is high, the family tends to have a high percentage of remote homologs. If the mean is low, the family tends to have a single dominant subfamily. When the query sequence is a good representation of the family consensus, the ICP and MACP traces are nearly identical. When the query sequence is poorly represented in the consensus, the MACP is not a good representation for the subfamily of the query. The ICP, however, remains valid as long as the criteria outlined in Figure 4 hold. These criteria are generally solid for any family with at least 30 members and they hold for some families with as few as 10 members.

In its original form, the NPLA algorithm required the query sequence to be compared with every sequence in UniProt. This was slow from a computational standpoint requiring up to 1 hour to process a single query. Since the distribution of the noise remains the same as long as the sample of noise is sufficient, a method that eliminates most noise, but does not eliminate the signal should lower the computational time without affecting the ultimate result. Such a method was developed based on an inverted index of trigrams. Detailed comparison studies showed that the noise could be reduced by 98% without changing the final result. Under these circumstances a processing time of 1 hour was reduced to 1 minute.

ICP conservation profiles can be rapidly generated for any query sequence without knowledge of domains or family relationships. The statistical validity of the traces is easy to assess. This approach provides a means for generating conservation profiles when the subfamily sample size is too small for the multiple alignment approach. Since the ICP is specific to the query sequence and it covers all the residues in the sequence, it is useful for studies that seek to correlate sequence derived features such as hydropathy or structure derived features such as normal modes (GNM) [23] with conservation. Studies are currently underway relating such features to the ICP using relative entropy measurements and Mahalanobis distances [24].

## Methods

### Database sources and histograms

The database for the studies in this paper contained data downloaded from the Pfam ftp site [25]. The data was downloaded on 02-Nov-2005. The database also contained a total of 2,345,429 entries from the UniProt database [16]. Exclusion of sequences shorter than 75 residues or longer than 1500 residues resulted in a final set of 2,128,677 sequences. This will be referred to as the SPT data set. The family memberships used in this paper were obtained from the Pfam-A.full file in the Pfam distribution. Pfam-A contained 8,183 families. This file also served as the source for the multiple alignments associated with each Pfam-A family.

The sequences in the SPT data set were parsed into a series of n-gram sets and n-gram pattern sets. N-grams are contiguous runs of n residues. N-gram patterns are contiguous runs of residues and wildcards (see below). Histograms reflecting the probability of a given n-gram were constructed for values of n ranging from 1 to 8. Similar histograms were constructed bracketing the range of n-gram patterns reported in this study.

### Consensus based conservation profiles

Conservation profiles were generated from multiple alignments using the Valdar-Thornton approach [3,26]. These profiles will be referred to as MACPs (multiple alignment conservation profiles). The mathematical basis for MACPs is presented in equations 1–4.

(1) $$Cons(i)=\frac{\sum_{j}^{N}\sum_{k>j}^{N}W_{j}W_{k}Mut(S_{j}(i),S_{k}(i))}{\sum_{j}^{N}\sum_{k>j}^{N}W_{j}W_{k}}$$

In this equation, *Cons(i) *represents the degree of conservation at *i*th position in a multiple alignment. It is a number that varies from a minimum of 0.0 to a maximum of 1.0. The total number of sequences in the multiple alignment is represented by *N*. *Mut(S*_*j*_*(i), S*_*k*_*(i)) *represents the score associated with a mutation at the *i*th position between the *j*th and *k*th sequences in the multiple alignment. The weights *W*_*j *_and *W*_*k *_associated with the *j*th and *k*th sequences are used to correct for sample skew. The mutation score *Mut(a,b) *associated with a mutation of amino acid *a *to amino acid *b *is obtained from a substitution matrix using equation 2. The substitution matrix used in this study was BLOSSUM62 [2].

(2) $$Mut(a,b)=\{\begin{array}{c} \frac{m(a,b)-\min(m)}{\max(m)-\min(m)}\\ \begin{array}{c} 0 \end{array} \end{array}\begin{array}{c} , \end{array}if\begin{array}{c} a \end{array},b\begin{array}{c} are\begin{array}{c} not\begin{array}{c} gap \end{array} \end{array} \end{array}$$

In this equation, *m(a,b) *represents the score obtained from the substitution matrix when residue *a *is replaced by residue *b *while *max(m) *and *min(m) *represent the highest and lowest substitution scores in the matrix. This has the effect of normalizing the scores in the substitution matrix to the interval from 0.0 to 1.0. The weights used to correct for sample skew are calculated using equation 3. In this equation *W*_*j *_is the weight associated with the *j*th sequence in the multiple alignment. *N *is the total number of sequences in the multiple alignment and *Dist(S*_*j*_, *S*_*k*_*) *is the evolutionary distance between the *j*th and the *k*th sequences.

(3) $$W_{j}=\frac{\sum_{k\neq j}^{N}Dist(S_{j},S_{k})}{N-1}$$

The evolutionary distance *Dist(S*_*j*_, *S*_*k*_*) *between the *j*th and *k*th sequences in a multiple alignment is defined by equation 4. In this equation, sequences that are close to each

(4) $$Dist(S_{j},S_{k})=1-\frac{\sum_{all\begin{array}{c} residues \end{array}}Mut(S_{j},S_{k})}{Num(aligned\begin{array}{c} positions \end{array})}$$

other from the evolutionary standpoint have small values for *Dist(S*_*j*_, *S*_*k*_*)*. The final MACP profile for each sequence was obtained by removing the non-residue positions in each sequence in the multiple alignment.

### Hierarchical clusters

The pairwise evolutionary distance between the members of each of the 120 Pfam-A families listed in Table 2 was determined using equation 4 from the previous section. A hierarchical cluster was developed for each of these families using the following procedure beginning with the root level: (1) find the two sequences with the greatest difference in evolutionary distance; (2) separate the level into two clusters based on proximity to these two sequences; (3) repeat the process recursively until each terminal node contains a single sequence; (4) reassign the terminal nodes one at a time if there is a preterminal cluster that is closer than their current parent cluster; (5) relink and reweight the tree after each reassignment; (6) continue this process until all terminal nodes are in optimal position. For each query sequence, identify the cluster of sequences at each level in the corresponding hierarchy that contains the query sequence. Generate an MACP from this set if the set contains 20 or more members.

**Table 2:** Pfam-A Seed Alignment Sources used in ICP-MACP Comparison

**S30 (N = 207)**		**S100 (N = 441)**		**S500 (N = 1,666)**		**S1000 (N = 1,931)**	
PF02160	PF07292	PF00778	PF03351	PF00215	PF01544	PF00038	PF00974
PF02262	PF07298	PF00812	PF03428	PF00251	PF01555	PF00079	PF01032
PF02288	PF07386	PF01190	PF03619	PF00317	PF01561	PF00239	PF01138
PF02509	PF07413	PF01199	PF03663	PF00328	PF01638	PF00285	PF01423
PF02783	PF07454	PF01491	PF03709	PF00410	PF01794	PF00348	PF01425
PF03068	PF07515	PF01500	PF03914	PF00464	PF02491	PF00437	PF01699
PF03173	PF07574	PF01874	PF03942	PF00466	PF02910	PF00462	PF02080
PF03206	PF07775	PF01946	PF04221	PF00636	PF03062	PF00481	PF02811
PF03215	PF07781	PF02005	PF04329	PF00650	PF03358	PF00482	PF02932
PF03409	PF07842	PF02076	PF05047	PF00781	PF03507	PF00498	PF03764
PF03612	PF07959	PF02589	PF05127	PF00846	PF04069	PF00682	PF03797
PF03676	PF08001	PF02709	PF06761	PF01056	PF04607	PF00690	PF04539
PF03762	PF08004	PF02758	PF07470	PF01065	PF05368	PF00844	PF07728
PF03829	PF08315	PF03140	PF07486	PF01409	PF07478	PF00899	PF07885
PF03964	PF08470	PF03350	PF08379	PF01421	PF08541	PF00948	PF08544

## Authors' contributions

Both JKV and XL contributed to this paper, and also read and approved the final manuscript.
